# Hypertriglyceridemia‐Induced Acute Pancreatitis Associated with Ruxolitinib for Hemophagocytic Lymphohistiocytosis: A Case Report

**DOI:** 10.1155/crom/7271334

**Published:** 2025-12-10

**Authors:** Sarah Medina, Leslie A. Ynalvez, Hyeon-Ju Ryoo Ali, Maria E. Cabanillas, Ihab Hamzeh, Salil Kumar, Nicolas Palaskas, Anita Deswal, Shaden Khalaf

**Affiliations:** ^1^ Department of Cardiology, The University of Texas MD Anderson Cancer Center, Houston, Texas, USA, mdanderson.org; ^2^ Department of Endocrine Neoplasia and Hormonal Disorders, The University of Texas MD Anderson Cancer Center, Houston, Texas, USA, mdanderson.org

**Keywords:** hyperlipidemia, hypertriglyceridemia, Jakafi, pancreatitis, ruxolitinib, triglycerides

## Abstract

Ruxolitinib is a Janus kinase inhibitor that has been associated with lipid abnormalities, including a 15% incidence of hypertriglyceridemia. We describe a case of a 37‐year‐old man with refractory T‐cell lymphoma treated with ruxolitinib for hemophagocytic lymphohistiocytosis (HLH). Following ruxolitinib use, the patient developed severe epigastric abdominal pain with elevated amylase, lipase, and triglycerides. This led to a suspicion of hypertriglyceridemia‐induced pancreatitis requiring an insulin infusion. Unfortunately, the patient experienced multiorgan failure and expired. While ruxolitinib has been associated with hypertriglyceridemia, severe lipid abnormalities, as observed in this case, are rare. Furthermore, assessing the incidence of severe hypertriglyceridemia in the setting of HLH is challenging, given that the disease itself contributes to elevated triglyceride levels. This case highlights the need for a more vigilant approach in monitoring lipid parameters when using ruxolitinib for HLH treatment, especially among patients with concomitant risk factors.

## 1. Introduction

Ruxolitinib (Jakafi) is a Janus kinase inhibitor used for the treatment of intermediate or high‐risk myelofibrosis (FDA approved 2011) [1], polycythemia vera with inadequate response to or intolerance of hydroxyurea (FDA approved 2014), and steroid‐refractory acute graft‐versus‐host disease (FDA approved 2019). The most common adverse effects include hematologic reactions, such as anemia, thrombocytopenia, and neutropenia because of its effects on growth factors responsible for hematopoiesis [2]. Ruxolitinib has also been associated with lipid abnormalities, including a 15% incidence of hypertriglyceridemia [2]. It is hypothesized that ruxolitinib may interrupt leptin signaling pathways, although the exact mechanism of which it affects triglyceride levels remains unknown [3]. We present a case report of hypertriglyceridemia‐induced acute pancreatitis associated with ruxolitinib use.

## 2. Case Presentation

A 37‐year‐old man with refractory T‐cell lymphoma, basal cell carcinoma, Wiskott–Aldrich syndrome, ulcerative colitis, and childhood immune thrombocytopenia initially presented to an outside institution for chemotherapy with gemcitabine and pembrolizumab as a bridge to Chimeric Antigen Receptor‐Natural Killer (CAR‐NK) cell therapy. Approximately 10 days after receiving gemcitabine and pembrolizumab, his triglycerides were noted to be elevated at 242 mg/dL (reference range < 150 mg/dL) with an unknown baseline. He had no reported history of hypertriglyceridemia or pancreatitis.

He was then admitted to our institution for planned CD70 CAR‐NK cell therapy. Following therapy, there was a concern for development of cytokine release syndrome (CRS) and hemophagocytic lymphohistiocytosis (HLH) (Table 1). Treatment with dexamethasone 20 mg IV every 12 h and ruxolitinib 10 mg via nasogastric tube every 12 h was initiated.

**Table 1:** Selected HLH labs.

**Laboratory results**	**Ferritin (reference range 30–400 ng/mL)**	**Fibrinogen (reference range 214–503 mg/dL)**	**Lactate Dehydrogenase (reference range 135–225 U/L)**	**Interleukin-2 Receptor (CD25) (reference range 175.3–858.2 pg/mL)**
Day 0^a^	67,659	140	5025	—
Day 13	87,143	150	1264	4592.1

^a^Day 0 defined as ruxolitinib initiation date.

**Figure 1:**
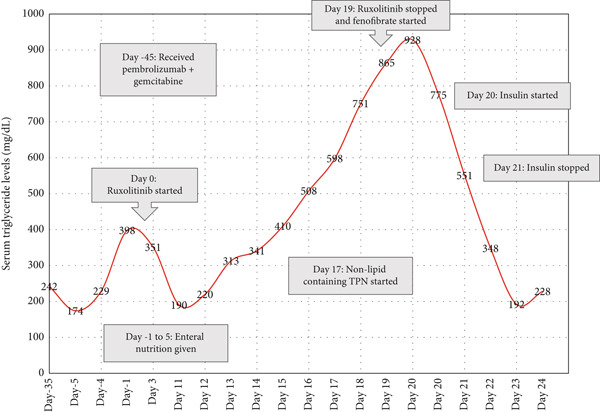
Serum triglyceride levels with ruxolitinib use (mg/dL).

Triglyceride levels were closely monitored during treatment because of elevated baseline values. The patient’s triglyceride levels markedly increased approximately 2 weeks after ruxolitinib initiation (Figure 1). Enteral nutrition with Novasource Renal formula (lipid containing formula) was discontinued on Day 5 after initiating ruxolitinib, and subsequent triglyceride levels were obtained in a fasting state as he remained nils per os (nothing by mouth) thereafter. He developed epigastric abdominal pain on day 8 of ruxolitinib therapy, which subsequently progressed in severity associated with abdominal distension and decreased bowel sounds over the next 12 days. Amylase level on onset of epigastric pain was 4.8 times the upper limit of normal and lipase level was 2.3 times the upper limit of normal (Table 2). Triglyceride trend across the clinical course is delineated in Figure 1. The overall course was concerning for triglyceride‐induced acute pancreatitis.

**Table 2:** Amylase and lipase levels.

**Laboratory results**	**Amylase level (reference range 28–100 U/L)**	**Lipase level (reference range 13–60 U/L)**
Day 9	487	139
Day 18	288	132
Day 20	369	121
Day 20	384	159

Contrast enhanced abdominal/pelvic computed tomography (CT A/P) revealed a large amount of free intraperitoneal air concerning for bowel perforation. No acute surgical intervention was performed for the perforation given his neutropenia. Based on endocrinology recommendations, ruxolitinib was discontinued and fenofibrate 48 mg daily via nasogastric tube was initiated. His triglycerides continued to uptrend to 928 mg/dL and he was transferred to the intensive care unit for management of suspected hypertriglyceridemia‐induced acute pancreatitis. He was started on a regular insulin (Humulin R) in sodium chloride (1 unit/mL) continuous infusion at a rate of 1 unit/h and dextrose 10% (D10W) infusion at a rate of 30 mL/h. Total parenteral nutrition (TPN) was held. Although his triglycerides did not reach the pancreatitis treatment goal of < 500 mg/dL, the insulin infusion was stopped the next morning because of hypoglycemia nonresponsive to dextrose infusion. His clinical course continued to deteriorate into multiorgan failure. With his critical state, multidisciplinary discussion with the patient’s family transitioned his code status to Do Not Resuscitate (DNR). He went into a pulseless electrical activity cardiac arrest and expired.

## 3. Discussion

The elevation in triglyceride levels was likely multifactorial and may have occurred in parts because of his HLH diagnosis, suspected hypertriglyceridemia even prior to ruxolitinib therapy, non‐lipid containing TPN use, or pembrolizumab therapy. Ruxolitinib therapy was initiated for HLH, which includes hypertriglyceridemia as part of the diagnostic criteria. The HLH‐2004 guidelines include a fasting triglyceride threshold of 285 mg/dL, and previous research has shown that most patients diagnosed with HLH exhibit hypertriglyceridemia [4, 5]. Although the mechanism is not fully understood, the release of cytokines and elevated tumor necrosis factor‐alpha levels seen in HLH may lead to decreased lipoprotein lipase activity and lipid metabolism [6].

The patient was initiated on TPN approximately 2 weeks after ruxolitinib initiation. Although his TPN formulation did not contain any lipids, there have been documented cases of hypertriglyceridemia resulting from the metabolic conversion of dextrose into fatty acids [7]. However, this seems less likely in this case, as the TPN was initiated after his triglyceride levels had already begun to rise, the dextrose concentration had not yet reached the target level, and the TPN was only administered for a few days.

The rise in triglycerides may have also been linked to other medications. Initially, the patient’s triglycerides were noted to be elevated after receiving pembrolizumab and gemcitabine. Pembrolizumab has been known to be associated with hypertriglyceridemia, with previous reports showing a variable onset of lipid abnormalities [8, 9]. Considering the longer half‐life elimination of pembrolizumab, it is possible that it contributed to the rise in triglyceride levels.

In addition to the present case report, previous studies have shown that ruxolitinib use is associated with lipid abnormalities, although severe hypertriglyceridemia is rare. The REACH1 trial assessed ruxolitinib for steroid‐refractory acute graft‐versus‐host‐disease and found that 12.7% of patients experienced any grade of hypertriglyceridemia, with 2.8% of patients experiencing Grade 3 or 4 effects [10]. The RESPONSE trial assessed ruxolitinib for polycythemia vera and found more patients in the ruxolitinib group experienced hypertriglyceridemia compared with the standard therapy group (20.9% vs. 6.3%). However, none of the patients experienced Grade 3 or 4 effects, and the researchers concluded that the elevations in triglyceride levels were not associated with clinical outcomes [11]. A previous case report by Watson et al. reported life‐threatening hypertriglyceridemia associated with ruxolitinib and sirolimus therapy, with a notable increase in serum triglycerides seen approximately 1 week after ruxolitinib initiation [12]. This timeline corresponds with the laboratory findings in the current report, in which triglycerides began to rise around Day 12. Furthermore, the rapid decline in triglyceride levels following ruxolitinib discontinuation strengthens the hypothesis that the drug played a role in the onset of hypertriglyceridemia, although initiation of fenofibrate also likely contributed to the triglyceride lowering.

## 4. Conclusion

Ruxolitinib is a commonly used agent and generally well‐tolerated. This case demonstrates that hypertriglyceridemia and pancreatitis may occur with its use. The incidence of ruxolitinib‐associated severe hypertriglyceridemia remains unclear, particularly in the setting of HLH. Given that HLH itself increases the risk of hypertriglyceridemia, it is possible that a synergistic effect may have occurred. The manufacturer recommends assessing lipid parameters 8–12 weeks after ruxolitinib initiation. However, more frequent monitoring may be warranted in the inpatient setting, especially among patients with pre‐existing lipid disorders or additional risk factors. This case supports the need for a more vigilant approach in monitoring for lipid abnormalities during ruxolitinib use in the setting of HLH.

## Ethics Statement

The case report was approved for publication by The University of Texas MD Anderson Cancer Center Institutional Review Board under approval number 2025‐0263‐MDACC.

## Consent

Written informed consent for publication has been obtained from the patient’s family/legal next of kin.

## Disclosure

All authors have approved the final version and submission of the manuscript.

## Conflicts of Interest

The authors declare no conflicts of interest.

## Author Contributions

Authors Sarah Medina and Shaden Khalaf contributed to the conceptualization and writing of the original draft. Authors Leslie A. Ynalvez, Hyeon‐Ju Ryoo Ali, Maria E. Cabanillas, Ihab Hamzeh, Salil Kumar, Nicolas Palaskas, and Anita Deswal contributed to the review and editing of the manuscript.

## Funding

No funding was received for this manuscript.

## Data Availability

The data that support the findings of this study are available on request from the corresponding author. The data are not publicly available due to privacy or ethical restrictions.
